# A Dynamic Visualization Tool of Local Trends in Heart Disease and Stroke Mortality in the United States

**DOI:** 10.5888/pcd19.220076

**Published:** 2022-09-08

**Authors:** Phong Le, Michele Casper, Adam S. Vaughan

**Affiliations:** 1 Division for Heart Disease and Stroke Prevention, Centers for Disease Control and Prevention, Atlanta, Georgia

## Abstract

Efforts in the US to prevent and treat cardiovascular disease (CVD) contributed to large decreases in death rates for decades; however, in the last decade, progress has stalled, and in many counties, CVD death rates have increased. Because of these increases, there is heightened urgency to disseminate high-quality data on the temporal trends in CVD mortality. The Local Trends in Heart Disease and Stroke Mortality Dashboard is an online, interactive visualization of US county-level death rates and trends for several CVD outcomes across stratifications of age, race and ethnicity, and sex. This powerful visualization tool generates national maps of death rates and trends, state maps of death rates and trends, county-level line plots of annual death rates, and bar charts of percentage changes. County-level death rates and trends were estimated by applying a Bayesian spatiotemporal model to data obtained from the National Vital Statistics System of the National Center for Health Statistics and US Census bridged-race intercensal estimates for the years 1999 through 2019. The Local Trends in Heart Disease and Stroke Mortality Dashboard makes it easy for public health practitioners, health care providers, and community leaders to monitor county-level spatiotemporal trends in CVD mortality by age group, race and ethnicity, and sex and provides key information for identifying and addressing local health inequities in CVD mortality trends.

**Summary**

**What is already known on this topic?**

Cardiovascular disease (CVD) death rates have decreased in recent decades. However, in the last decade CVD death rates in many counties increased. Dissemination of local CVD trend data is critical to address increasing mortality.

**What is added by this report?**

This report introduces the Local Trends in Heart Disease and Stroke Mortality Dashboard, an online, interactive visualization of county-level death rates and trends for several CVD outcomes across stratifications of age, race and ethnicity, and sex.

**What are the implications for public health practice?**

This dashboard makes it easy for public health practitioners, health care providers, and community leaders to identify and address local health inequities in CVD mortality trends.

## The Importance of Documenting Local Trends in CVD Mortality

Declines in cardiovascular disease (CVD) mortality in the US have been recognized as 1 of the 10 great public health achievements of the 20th century (1). These declines represent decades of successful efforts to improve CVD prevention and treatment, including decreases in smoking, increases in blood pressure control, and medical advances in early detection and treatment (2). However, these declines were not equally shared across geography and demographic groups (3). Counties in the southern US and Black adults across the US experienced less favorable trends, contributing to the marked geographic and racial disparities observed today (3–5).

CVD death rates have recently plateaued or begun to increase. National declines have stagnated in the last decade. For many counties in states across the US, CVD death rates, including those from heart disease and stroke, have increased (6–8). Unlike the highest CVD death rates, which are concentrated in the southern US, increases in CVD death rates are widespread and occur in counties in almost all US states (7–9). Additionally, these increases are more prevalent among adults aged 35 to 64 years than among adults aged 65 years or older, and are observed across race, ethnicity, and sex.

Because of these trends and the marked geographic and demographic variation, the dissemination of high-quality local data on the temporal trends in CVD mortality assume heightened urgency. Public health practitioners, clinicians, and community leaders can use these data to inform policy and program decisions (10). For example, local data could be instrumental in prioritizing prevention efforts among demographic groups in places with increasing CVD death rates. Likewise, local CVD mortality data could reveal racial, ethnic, and geographic disparities masked by national data. To make county-level CVD death rates and trends more readily available and easily visualized, we created the Local Trends in Heart Disease and Stroke Dashboard (https://www.cdc.gov/dhdsp/maps/hd-stroke-mortality-dashboard.htm). The dashboard is an online, interactive visualization of death rates and trends for several CVD outcomes by age group, racial and ethnic group, and sex.

## Spatiotemporal Models of CVD Death Rates and Trends

The Local Trends in Heart Disease and Stroke Mortality Dashboard reports death rates and trends for 4 types of CVD: heart disease, coronary heart disease (CHD), heart failure, and stroke. We obtained county-level data for all deaths for the years 1999–2019 from the National Vital Statistics System of the National Center for Health Statistics. This period corresponds to the implementation of the *International Classification of Diseases, Tenth Revision* (ICD-10) (11). We used US Census bridged-race intercensal estimates for population data. Cause of death was defined according to the underlying cause of death listed on the death certificate and classified according to the following ICD-10 codes: CVD, I00–I99; heart disease, I00–I09, I11, I13, and I20–I51; CHD, I20–I25; and stroke, I60–I69. Death attributable to heart failure was defined as deaths for which any listed cause of death included “heart failure” (ICD-10 code I50) and “heart disease” (as defined above) was the underlying cause of death.

We used a Bayesian spatiotemporal model to estimate county-level CVD death rates by age group (ages 35–64 and ≥65 years), race and ethnicity (non-Hispanic American Indian/Alaska Native, non-Hispanic Asian/Pacific Islander, non-Hispanic Black, Hispanic, and non-Hispanic White), and sex (male and female) (12,13). Briefly, by accounting for correlation across space, time, and demographic groups, Bayesian spatiotemporal models can generate precise, reliable rates, even in the presence of small case counts and small populations. We fit these models with a Markov chain Monte Carlo algorithm. All death rates were age‐standardized to the 2010 US population by using 10‐year age groups. These models have been used extensively to document spatiotemporal trends in CVD mortality, including deaths due to stroke, heart disease, and heart failure (3–5,7–9,13–16).

To quantify the temporal trends, we estimated total percentage change in death rates by using log‐linear regression that included all years within each interval. Using relative change instead of absolute change allowed the comparison of results across outcomes and demographic groups. These comparisons would not be possible if absolute change were used because of large variation in death rates across outcomes and demographic groups. Our use of log-linear regression also permitted all rates to inform estimates of percentage change, which is not the case when calculating percentage change by using simple differences in rates between the beginning and end of each period.

To ensure that we reported precise rates only in sufficiently large populations, data for a demographic group within a county were suppressed if that group’s population in the given county in 2019 was fewer than 500 people and the death rates for all years were not reliable (ie, the width of the credible interval was smaller than the point estimate). This definition for suppression has been used extensively in studies that report county-level CVD mortality (5,8,9,14–16). Using this definition, different counties were suppressed by age, race and ethnicity, and sex for each outcome. However, within each demographic group, the same counties were suppressed for each outcome across all years.

We performed all statistical modeling in R (R Foundation for Statistical Computing) and used user-developed code. Additional details about the statistical analysis for these models are available in the dashboard.

## Efficient Storage of Death Rate and Trend Data

The task of visualizing CVD death rates and trends across distinct spatial, temporal, and demographic strata required more than 75 million data points and demonstrated a need to efficiently store data. Increased efficiency in storage allowed the dashboard to speedily query data and to optimize load times. Data were stored in a relational database by using the second normal form (2NF) (17). Every combination of age, race and ethnicity, and sex had a distinct line of data that allowed for parameter-based query. The use of 2NF allowed for data points with repeated values to be stored separately and called only when needed. For example, each US county has an associated Federal Information Processing Standard (FIPS) code. When the FIPS code is selected, the associated county, state, and geographic data (ie, data that remain constant) can be saved in a separate table instead of repeating these data points with the varying rate and trend data. As a result, the storage required for the 75 million data points in the database decreased from more than 5 gigabytes to fewer than 0.5 gigabytes (90% improved efficiency).

## Key Visualizations and Features

**Figure 1:**
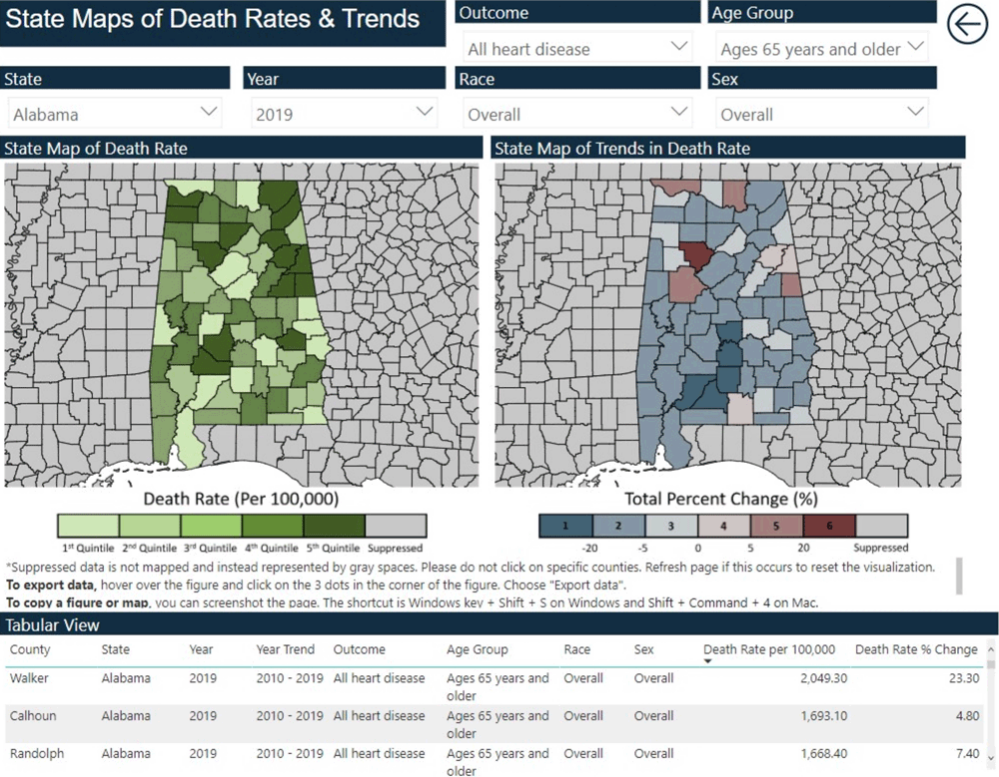
Maps showing the full interface of the Local Trends in Heart Disease and Stroke Mortality Dashboard.

The Local Trends in Heart Disease and Stroke Mortality Dashboard is an intuitive, self-guided, online dashboard that provides high-quality data on trends in local CVD mortality to public health practitioners, clinicians, and community leaders for use in informing policy and program decisions. When first visiting the dashboard, users are shown a curated landing page that briefly describes the dashboard and allows for navigation to views at the national, state, and county levels. This navigation was designed to allow users to immediately select the geographic level of interest. Each view has interactive visualizations that automatically update according to the combination of user selections that stratify by geography, period, disease outcome, age group, race and ethnicity, and sex (Figure 1). Each view also includes a table that allows users to examine line listings of the displayed data. 

**Figure 2:**
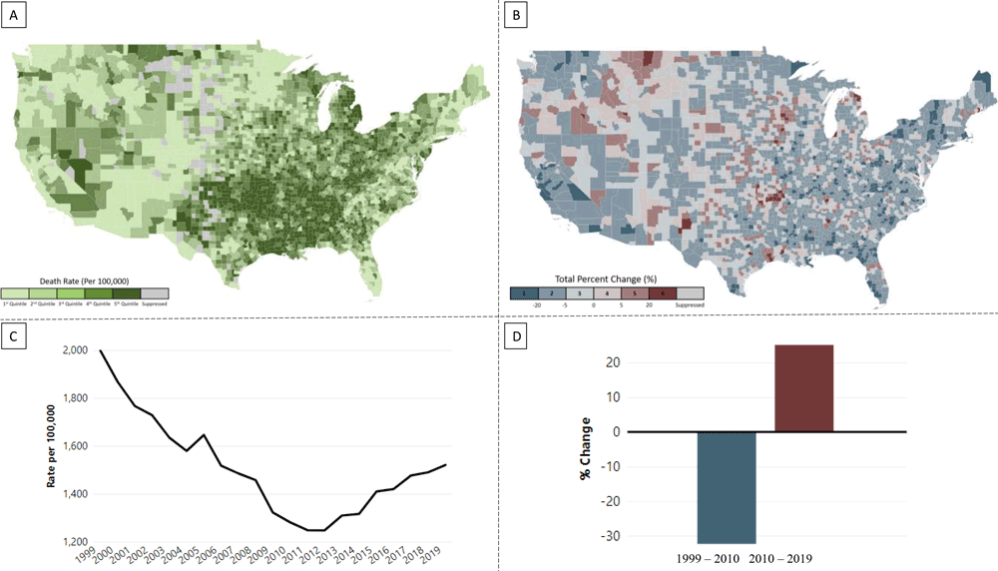
Example of visualizations of death rates for all heart disease by county among population aged ≥65 years, all races and ethnicities, and both sexes in the Local Trends in Heart Disease and Stroke Mortality Dashboard. A, National map of death rates, 2019. B, National map of trends in death rates, 2010–2019. C, Annual death rates in Alpena County, Michigan, 1999–2019. D, Trends in death rates in Alpena County, Michigan, 1999–2010 and 2010–2019.

The national and state views include maps of county-level death rates and trends (Figure 2A and Figure 2B). Maps of death rates provide monochromatic visualization of county-level death rates for a selected year (1999 through 2019). Trend maps use a divergent color scheme to visualize county-level trends in death rates for either the decade of 1999–2010 or 2010–2019. All maps allow users to hover over counties to see county-specific death rates and trends. National maps of death rates and trends are displayed separately, while state maps allow for a side-by-side comparison of death rates and trends.

The county-level view includes line graphs of annual death rates and bar charts of percentage change for the period 1999–2019 (Figure 2C and Figure 2D). Unlike the national and state views, county-level views display a single county’s data for all years. The line plots enable the user to see annual changes and overall temporal trajectory of death rates in each county. The bar charts offer a summary of the magnitude of percentage change for 2 periods (1999–2010 and 2010–2019).

## Usability Design and Feedback

This dashboard was designed by using the PowerBI platform (Microsoft Corp). PowerBI provides a point-and-click solution for dashboard creation, which allows for development by all programming skill levels. Furthermore, PowerBI contains many features that increase its accessibility to users as defined by Section 508 of the Rehabilitation Act of 1973 (18), including built-in keyboard shortcuts, approved color schemes, and the ability to specify alternative text.

Key partners in the National Center for Chronic Disease Prevention and Health Promotion at the Centers for Disease Control and Prevention were invited to provide feedback on the dashboard’s ability to convey intuitive visualizations, layout preferences, and accessibility. The feedback formed the basis for the implementation of key decisions for the dashboard, such as the decision to arrange views by geographic scope (national, state, county). Views were optimized so that users could use the visualizations in a way that best suited them. For example, users primarily interested in data at a national level requested that the national maps include Hawaii and Alaska to represent a complete national view. Users primarily interested in data at the state level preferred the side-by-side view to directly compare the geographic patterns of CVD death rates and trends.

## Examples of How the Dashboard Can be Used

The Local Trends in Heart Disease and Stroke Mortality Dashboard can benefit public health and community organizations addressing CVD mortality in numerous ways. The county-level visualizations enable users to identify counties with high or increasing mortality and tailor CVD prevention and treatment programs and policies to the needs of those communities (10,19,20). Given the spatiotemporal nature of the dashboard, areas that may benefit from efforts to improve cardiovascular health may be defined according to worsening temporal trends in CVD mortality rather than on high death rates alone. Additionally, data from the dashboard may be downloaded, allowing users to combine county-level CVD mortality trend data with county-level measures of local social, structural, and economic factors, to better understand the context for the observed trends (10,20). Furthermore, the ability to stratify trend data by demographic variables, such as race and ethnicity and sex, and to download all data and figures allows organizations to tailor CVD prevention programs and policies to the needs of key demographic groups in specific locations. Finally, the CVD surveillance data in this dashboard may be updated to include additional years of data or to reflect other notable county-level data.

## Summary

In light of widespread county-level increases in CVD death rates, there is a heightened urgency to make high-quality local-level trend data and maps easily available to public health practitioners, health care providers, and community leaders. The Local Trends in Heart Disease and Stroke Mortality Dashboard is an online interactive data visualization tool that makes it easy to monitor county-level spatiotemporal trends in CVD mortality by age group, racial and ethnic group, and sex. Using these data, the dashboard can provide key information for identifying and addressing local-level health inequities in CVD mortality trends.
